# A case series of therapy-related leukemias: A deadly ricochet

**DOI:** 10.1016/j.lrr.2023.100382

**Published:** 2023-07-28

**Authors:** Ronit Juthani, Ashish Ranjan Singh, Debdatta Basu

**Affiliations:** a Department of Internal Medicine, Saint Vincent Hospital, Worcester, MA 01608, United States; b Department of Pathology, JIPMER, Puducherry, India

**Keywords:** Therapy-leukemia, Myeloid, Chemotherapy

## Abstract

Therapy-related leukemias(t-leukemia) are late complications arising from chemotherapy and radiotherapy. t-leukemia have a poor prognosis and are more difficult to treat compared to de novo leukemias. The authors present three cases of t-leukemia seen in our hospital in a three year period and discuss new updates concerning the treatment of t-leukemia.

## Abstract (author highlights)

Case 1: A 62-year-old woman previously who developed acute myelomonocytic leukemia (AML-M4) three years after etoposide-based chemotherapy.
Case 2: A 9-year-old boy who developed acute myeloid leukemia without maturation (AML-M1) after receiving cisplatin, adriamycin, and etoposide chemotherapy.
Case 3: 1-year-old child who developed common B-cell acute lymphoblastic leukemia (B-ALL) after completing chemotherapy.

## 1 Background

The 2022 WHO leukemia classification lists therapy-related myeloid neoplasm(t-MN) as a distinct entity that should be approached differently [1]. Subclassified into t-MDS and t-AML, they are considered late complications of chemotherapy and radiotherapy. Therapy-related acute lymphoblastic leukemias, while not having the same privileges in the WHO classification as myeloid neoplasms, are not uncommon and share similar characteristics to therapy-related myeloid neoplasms [2]. Alkylating agents and topoisomerase II inhibitors are the most common agents involved in therapy-related leukemias(t-leukemia). Whole-body irradiation preceding allogeneic transplantation has also been found to be a responsible factor [3]. Therapy-related neoplasms constitute roughly 10–20% of newly diagnosed myeloid neoplasms, but the incidence is expected to rise due to greater accessibility of chemotherapy with better survival among patients. The median age of diagnosis of t-MN is 64 years, but the disease can occur at any age. The most common primary malignancies associated with therapy-related myeloid neoplasms include breast cancer and Non-Hodgkin lymphoma [4,5]. Therapy-related neoplasms tend to carry a poorer prognosis and are associated with a more significant proportion of high-risk cytogenetics than de novo leukemias [6]. Emerging research has also implicated acquired mutations in hematopoietic stem cells as a potential cause, likely explaining the varying susceptibility to cytotoxic chemotherapy and radiation [7]. Another critical challenge is differentiating secondary neoplasms, which are related to cancer susceptibility syndromes in patients and can only be discriminated from therapy-related neoplasms based on an antecedent cytotoxic therapy history [8]. While historically treated similarly to de novo leukemias, the last few years have seen the emergence of new regimens that have demonstrated better results than standard chemotherapy. The recent developments around t-leukemia revitalize the need for discussion on the topic. Here we present three cases of t-leukemia seen in our hospital in a three year period and discuss the essential points to be kept in mind while approaching a case of leukemia associated with prior therapy.

## 1.1 Case 1

**Fig. 1:**
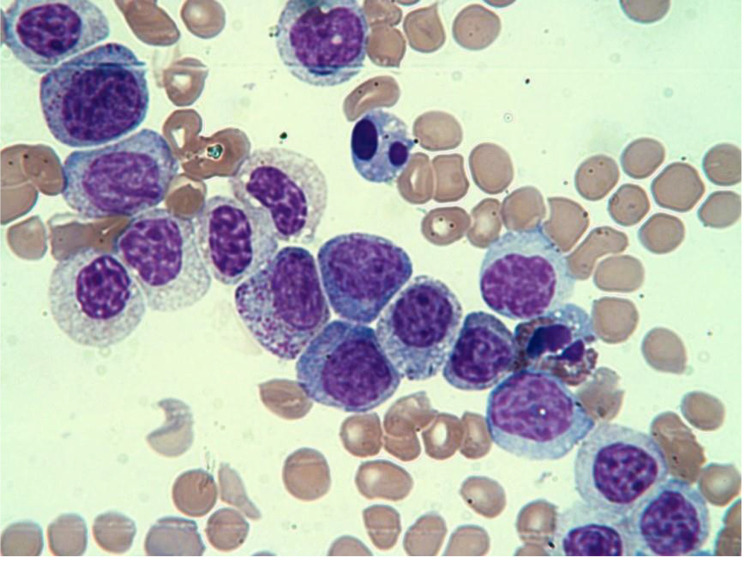
Peripheral smear showing myeloblasts, monoblasts, and promonocytes (Giemsa × 400).

A 62-year-old lady was diagnosed in 2012 with stage 3 Carcinoma Breast. She underwent a modified radical mastectomy followed by Etoposide-based chemotherapy), following which she went into complete remission from the disease. She did not receive any radiation therapy. She returned three years to the hospital with severe pallor and was found to have deranged blood counts. Her hemoglobin was 55 g/L, her total leucocyte count was 34.5 × 10^9^/L and her platelet count was 15 × 10^9^/L. Peripheral blood smear revealed the presence of 85% blast population with monocytoid morphology [Fig. 1]. Bone marrow aspirate and biopsy revealed blasts with suppressed trilineage hematopoiesis. These blasts were positive for Sudan Black B, Non-specific esterase, and Myeloperoxidase. Flow cytometry showed the blasts to be positive for CD34, HLA-DR, MPO, and CD14. Cytogenetic analysis showed 46,XX,t(X:10)(p10 or p21;p10 or p12)[17]/46,XX[3]. She was diagnosed with Acute Myelomonocytic Leukemia (AML-M4) and started on Azacytidine therapy. Unfortunately she succumbed to the disease in May 2015 due to sepsis related to cytopenia.

## 1.2 Case 2

**Fig. 2:**
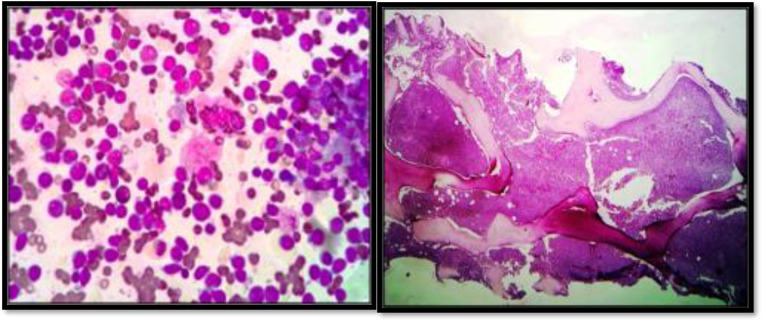
(a) BM aspirate showing myeloblasts (b) BM biopsy showing trilineage suppression and replacement by blasts.

A 9-year-old boy was diagnosed in a cervical lymph node biopsy with stage 4 metastatic neuroblastoma with a primary in the adrenal gland. He received eight cycles of Cisplatin, Adriamycin, and Etoposide chemotherapy. An I-131 MIBG scan done post-chemotherapy showed residual disease in the abdomen and neck. He was planned for palliative therapy with cis-retinoic acid but defaulted and returned two months later (two and a half years since the primary diagnosis) with pallor and hepatosplenomegaly. Complete blood count showed a hemoglobin of 80 g/L, a total leucocyte count of 10 × 10^9^/L, and a platelet count of 51 × 10^9^/L. Peripheral blood smear and bone marrow aspirate showed myeloblasts [Fig. 2(a)] positive for Sudan Black. Bone marrow biopsy revealed a hypercellular marrow with near total replacement by blasts [Fig. 2(b)] that were positive for CD34, MPO, CD 117, and negative for TdT. A small focus of residual neuroblastoma was also seen in the biopsy. Flow cytometry was positive for CD117, CD13, CD33, CD34, and HLA-DR. Cytogenetics showed 46XY t(11;19) (q23;q23), t(21;21) (q10;q10). A final diagnosis of therapy related acute myeloid leukemia without maturation was made, and the patient was started on daunorubicin and cytarabine. He developed neutropenia and pneumonia and succumbed during the second week of therapy. This case has also been published previously as a case report [9].

## 1.3 Case 3

**Fig. 3:**
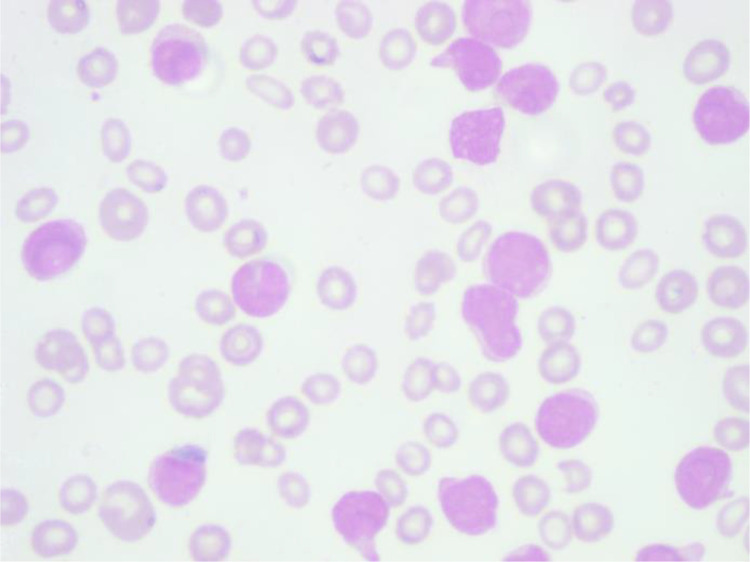
Peripheral smear showing leucocytosis with the presence of lymphoblasts (Leishman × 400).

An one-year-old child was diagnosed with retinoblastoma. Enucleation of the eye was done to remove the tumor, and the patient completed chemotherapy, the details of which are unavailable. Following this treatment, the patient was in remission of retinoblastoma. The patient returned four years later with abnormal blood counts. The complete blood count at admission showed a hemoglobin of 43 g/L, a total leucocyte count of 86 × 10^9^/L, and a platelet count of 35 × 10^9^/L. Peripheral blood smear showed the presence of 83% blasts which showed block positivity on Periodic acid Schiff (PAS) stain [Fig. 3]. Bone marrow aspiration and biopsy showed a predominant lymphoblast population. Immunohistochemistry and flow cytometry done revealed the blasts to be positive for CD34, CD79a, CD10, CD19, CD22, TdT, and HLA-DR and negative for myeloid or T-cell markers. Cytogenetics was, however, normal. The patient was diagnosed with common B-cell acute lymphoblastic leukemia (B-ALL) and started on BFM protocol. The patient went into remission and is currently on follow-up.

## 2 Discussion

There has been an improved survival and cure of many malignancies and it is imperative that more attention should now be devoted to minimizing the adverse delayed effects of chemotherapy and radiation to survivors, including the development of second malignancies. It must be understood that while a significant chunk of secondary t-leukemia has been attributed to breast cancer and lymphoma, all other cancers and their treatment are equally likely to lead to t-leukemia. While much literature is not available on therapy-related lymphoblastic leukemias as much is known on therapy-related myeloid neoplasms, it has primarily been found to have similar characteristics, including the involvement of similar etiologic agents, similar clinical presentations, similar cytogenetics, and a similarly worse prognosis compared to its de novo counterpart [2]. Alkylating agents have been the major contributing agent to t-leukemias and have a latency period of 5–10 years before the leukemia is diagnosed. Topoisomerase II inhibitors have a much shorter latency period of 1–5 years but do not cause as much t-leukemias as alkylating agents [10]. This latency period is more extended for patients with secondary leukemia who have not received chemotherapy [11]. T-leukemias has often been considered a disease of the elderly population [4]. It is important to understand, however, that t-leukemias can also occur in children, as seen in two of out three cases. Hence, it is essential that children who have received chemotherapy be monitored with regular blood count and symptom assessment. Compared with de novo leukemias, t-leukemias tend to have increased hemoglobin, decreased white blood cell and platelet count, and decreased percentage of blasts in peripheral blood and bone marrow [6]. This is important to remember since lower WBC levels can sometimes be at sub-leukemic and aleukemic levels, and it is still crucial that t-leukemia be kept as a differential in these cases. Unfortunately, immunohistochemistry and flow cytometry have been unable to distinguish between t-leukemias and other secondary and de novo leukemias; the only diagnostic clue is an antecedent history of chemotherapy.

Cytogenetics represents an interesting conundrum concerning t-leukemias. While the favorable, intermediate, and unfavorable cytogenetics remains the same for t-leukemias and de novo leukemias, the proportion of the population having unfavorable cytogenetics is way more significant in t-leukemias compared to de novo leukemias [6]. This observation has given rise to two parallel hypotheses regarding the origin of t-leukemias. One states that t-leukemias are caused due to the genotoxic effect of cytotoxic agents, while the other states that t-leukemias arise due to a clonal selection of genetically aberrant cells due to its relative resistance to cytotoxic agents [7]. Nevertheless, when patients with normal karyotypes were compared, patients with t-leukemias had a worse prognosis and overall survival than de novo leukemias [12]. The etiological agent may also determine the aberration that is seen: the use of alkylating agents is associated with monosomy of chromosomes 5 and 7, while the use of topoisomerase inhibitors is associated with balanced translocations involving 11q23 and 21q22 [13]. Unfortunately in none of the three cases could we do additional molecular studies as we did not have access to such investigations during the time of the study.

Most centers in India still have to rely on the traditional de novo leukemia treatment, which has not been as effective. To date, the only agent explicitly approved for t-leukemias is CPX-351, a liposomal preparation of daunorubicin and cytarabine approved for the treatment of therapy-related myeloid neoplasm [14]. For lymphoblastic leukemias, the treatment is usually similar to de novo leukemia [2]. Many new treatment options may soon get approved, with many agents currently in phase 3 clinical trials. The pace, however, remains slow for lymphoblastic leukemias. Besides, for patients who are eligible for hematopoietic stem cell transplantation(HSCT), HSCT may improve the patient's overall survival and relapse-free survival period [15].

The common theme binding the three cases we presented was the treatment with etoposide. This remains the single most important detail when diagnosing a case of t-leukemia. Other details may show variation but any history of chemotherapy should arouse a reasonable suspicion of therapy-related leukemia. It is also very important to have a cytogenetic analysis done, since they carry a summative prognostic effect. Our third case shows that a normal karyotype usually carries a better prognosis. While ideally treated in the same way as de novo leukemias, T-leukemias still portend a worse prognosis. Hence, strict regular blood count monitoring should be done after chemotherapy to ensure that an early diagnosis is possible.

## Funding

This research did not receive any specific grant from funding agencies in the public, commercial, or not-for-profit sectors.

## Declaration of Competing Interest

The authors declare that they have no known competing financial interests or personal relationships that could have appeared to influence the work reported in this paper.
